# Wrangling Real-World Data: Optimizing Clinical Research Through Factor Selection with LASSO Regression

**DOI:** 10.3390/ijerph22040464

**Published:** 2025-03-21

**Authors:** Kerry A. Howard, Wes Anderson, Jagdeep T. Podichetty, Ruth Gould, Danielle Boyce, Pam Dasher, Laura Evans, Cindy Kao, Vishakha K. Kumar, Chase Hamilton, Ewy Mathé, Philippe J. Guerin, Kenneth Dodd, Aneesh K. Mehta, Chris Ortman, Namrata Patil, Jeselyn Rhodes, Matthew Robinson, Heather Stone, Smith F. Heavner

**Affiliations:** 1 Department of Public Health Sciences, Clemson University, Clemson, SC 29634, USA; khowar7@clemson.edu (K.A.H.); sheavner@c-path.org (S.F.H.); 2 Center for Public Health Modeling and Response, Clemson University, Clemson, SC 29634, USA; 3 Critical Path Institute, Tucson, AZ 85718, USA; jpodichetty@c-path.org (J.T.P.);; 4 Centers of Disease Control and Prevention, Atlanta, GA 30329, USA; 5 Tufts University School of Medicine, Tufts University, Medford, MA 02155, USA; 6 Division of Pulmonary, Critical Care and Sleep Medicine, University of Washington, Seattle, WA 98195, USA; 7 IR Research & Academic Systems, University of Texas Southwestern, Dallas, TX 75390, USA; 8 Society of Critical Care Medicine, Mount Prospect, IL 60056, USA; 9 National Institutes of Health National Center for Advancing Translational Sciences (NCATS), Rockville, MD 20850, USA; 10 Infectious Diseases Data Observatory (IDDO), Nuffield Department of Medicine, University of Oxford, Oxford, Oxfordshire OX3 LF, UK; 11 Department of Emergency Medicine, Advocate Christ Medical Center, Oak Lawn, IL 60453, USA; 12 Department of Medicine, Emory University, Atlanta, GA 30322, USAjeselyn.rhodes@emory.edu (J.R.); 13 Institute for Translational and Clinical Science, University of Iowa, Iowa City, IA 52242, USA; 14 Brigham and Women’s Hospital, Boston, MA 02115, USA; npatil@bwh.harvard.edu; 15 Division of Infectious Diseases, Johns Hopkins University, Baltimore, MD 21205, USA; 16 US Food and Drug Administration, Silver Spring, MD 20993, USA; 17 Department of Biomedical Sciences, University of South Carolina School of Medicine Greenville, Greenville, SC 29605, USA

**Keywords:** clinical research, real-world data, factor selection, least absolute shrinkage and selection operator (LASSO) regression

## Abstract

Data-driven approaches to clinical research are necessary for understanding and effectively treating infectious diseases. However, challenges such as issues with data validity, lack of collaboration, and difficult-to-treat infectious diseases (e.g., those that are rare or newly emerging) hinder research. Prioritizing innovative methods to facilitate the continued use of data generated during routine clinical care for research, but in an organized, accelerated, and shared manner, is crucial. This study investigates the potential of CURE ID, an open-source platform to accelerate drug-repurposing research for difficult-to-treat diseases, with COVID-19 as a use case. Data from eight US health systems were analyzed using least absolute shrinkage and selection operator (LASSO) regression to identify key predictors of 28-day all-cause mortality in COVID-19 patients, including demographics, comorbidities, treatments, and laboratory measurements captured during the first two days of hospitalization. Key findings indicate that age, laboratory measures, severity of illness indicators, oxygen support administration, and comorbidities significantly influenced all-cause 28-day mortality, aligning with previous studies. This work underscores the value of collaborative repositories like CURE ID in providing robust datasets for prognostic research and the importance of factor selection in identifying key variables, helping to streamline future research and drug-repurposing efforts.

## 1. Introduction

Data-driven approaches to clinical research are necessary for understanding and effectively treating infectious diseases, including COVID-19 [1,2,3]. Data and research create avenues for identification of treatments, efficient distribution of resources, and dissemination of key results throughout the medical community [3]. However, due to disease rarity or ongoing and dynamic outbreaks, clinical data are often limited or closed-source, restricting information about potentially effective treatments [3,4]. Given such challenges, innovative methods to facilitate the organized, accelerated, and collaborative use of data must be a high priority.

Effective and timely treatment of infectious diseases is hindered by both features of the diseases themselves and barriers to drug development [5]. Many of the most challenging infectious diseases are either rare or emerging/re-emerging, posing problems for drug developers, such as a lack of capacity for clinical trials [5,6] or unrealistic time constraints on a process that takes years [7,8]. For example, amebic meningoencephalitis and infections with mycobacterium abscessus are too rare for companies to devote resources to drug development [9,10]; meanwhile, other infectious diseases, such as COVID-19, show rapid emergence coupled with persistent evolution, leaving little time for effective de novo drug development [11]. Drug repurposing may be an alternative that can accelerate development and approval of safe and effective treatments for difficult-to-treat diseases by leveraging real-world data and clinical research on “off-label” use of drugs developed and/or approved for other indications [12]. Drug repurposing may be less expensive, less time-consuming, and safer, as approved drugs have already gone through extensive safety testing, and many preclinical and early phase clinical trials may not be necessary [7,8,13]. Drug repurposing, rather than traditional de novo drug discovery and development, provided the first treatments for COVID-19 [14] and may be the most promising approach for identification of effective treatments for other difficult-to-treat infectious diseases.

However, despite considerable investigation into drug repurposing for COVID-19, as well as some success, there are persistent limitations [15]. Data-quality issues present one problem that has plagued research thus far, including poor study design, unreliable data sources, and inefficient data sharing [15,16]. As a result of data-quality issues, there has been a push for more data-driven approaches, with electronic health record (EHR) data identified as a promising source for generating hypotheses focused on repurposing [5,7,17]. Specifically, limitations may be resolved through collaborative and systematic approaches [5,7,18]. Organized data that are available in an open-source, publicly available model have been put forth as a key avenue for improved quality, collaborative idea sharing across the scientific and medical community, and a necessity in the face of COVID-19 and other difficult-to-treat infectious diseases [4,5,18,19].

In this context, machine-learning and feature-selection methods have emerged as essential tools in addressing the complexities of clinical data analysis. These techniques enable researchers to analyze vast and multidimensional datasets, uncovering patterns and relationships that may not be immediately evident. Considerations around the interpretability of these models are critical in clinical settings, where transparency and trust in decision-making processes are paramount. By balancing predictive accuracy with model explainability, these approaches provide actionable insights that can guide the prioritization of interventions and optimization of treatment strategies. This is especially valuable in drug repurposing, where identifying key predictors of treatment outcomes can accelerate the development of effective therapeutic solutions for challenging infectious diseases. An example of feature selection methodology is least absolute shrinkage and selection operator (LASSO) regression, which is a tool that has been widely used in various settings, such as disease risk prediction [20] and gene expression data analysis [21], as well as more specific areas, like intraductal papillary mucinous neoplasm of the pancreas [22], sepsis [23], and liver cancer [24], among others. Here, a predictor variable’s regression coefficient is constrained, such that those with the least influence or redundancy with other variables are shrunk to zero and excluded from the model [25]. Therefore, the most prognostic variables are retained within a model with minimized prediction error [25,26]. An additional advantage of LASSO regression is its interpretability, as the method not only identifies key variables but also provides insight into their relative importance in predicting the outcome. For example, LASSO regression has been used as a model for predicting COVID-19 severity [27]. However, despite the promise of such methodology for clinical research and assessing outcomes, limitations around data continue to persist, including the need for data from a variety of settings and collaborative databases to effectively aid in accelerating research in real-world settings [27].

As a collaborative, internet-based repository, CURE ID (https://cure.ncats.io/home, accessed on 8 July 2024) offers a potential approach for accelerating drug-repurposing research in difficult-to-treat infectious diseases, including COVID-19. Databases such as CURE ID can provide a de-identified dataset that is focused on priority variables and is readily available for investigators to perform drug-repurposing research and exploration. The purpose of the present study was to apply a data-driven approach to assess the usefulness of collaborative repositories, such as CURE ID, as resources for research on treatments for difficult-to-treat infectious diseases. Specifically, this research seeks to explore whether these data-driven repositories can effectively identify the variables most predictive of outcomes, supporting the potential for streamlined, collaborative research approaches to improve treatment identification in real-world settings. This study utilized COVID-19 data from six large US-based health systems to assess the utility of disease indicators (i.e., demographics, comorbidities, treatment, and laboratory measurements) from these data for an outcome of all-cause mortality within 28 days of hospitalization.

## 2. Materials and Methods

### 2.1. Data Source

Under the auspices of the CURE ID program and supported by a grant from the US Department of Health and Human Services’ Patient-Centered Outcomes Research Trust Fund, the CURE Drug Repurposing Collaboratory (CDRC) and the Society of Critical Care Medicine (SCCM) Discovery Critical Care Research Network partnered with eight healthcare institutions to obtain datasets of patients hospitalized with COVID-19, including demographics, vital signs, laboratory tests and measures, levels of oxygen support, and comorbidities. A full list of predictors in the datasets is provided in Table 1.

### 2.2. Data Analysis

**Figure 1:**
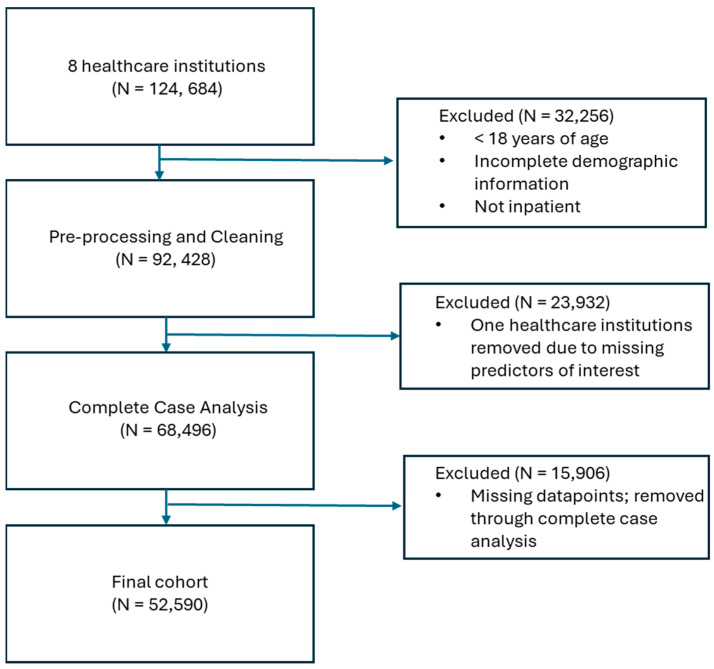
Flowchart showing how patients were eliminated from inclusion in the model.

A least absolute shrinkage and selection operator (LASSO) regression was utilized to examine the predictors of interest. It was trained on the data, with 28-day all-cause mortality (a binary variable where 0 = alive and 1 = dead) as the outcome of interest. The cohort was divided into sets of data meant for training and testing the model, where 4 sites were utilized for training, and 2 were randomly selected for testing (Figure 1). All predictors were entered into the model simultaneously. Laboratory predictor variables were minimum, median, mean, and maximum values for leukocyte count, monocyte count, lymphocyte count, eosinophil count, basophil count, hematocrit level, hemoglobin level, platelet count, total bilirubin, aspartate aminotransferase (AST) level, alanine aminotransferase (ALT) level, serum creatinine level, a race-neutral calculation of estimated glomerular filtration rate (eGFR) [28], respiratory rate, heart rate, temperature, and oxygen saturation through pulse oximetry (SpO2) captured during the first 48 h upon admission. We included age; race; sex; body mass index (BMI); and comorbidities of human immunodeficiency virus (HIV), chronic lung disease, chronic kidney disease, cardiovascular disease, and diabetes. In addition, we included the level of oxygen support supplied, with categories of no oxygen administered; oxygen in the form of masks, cannulas, or positive airway pressure; and mechanical ventilation. These were categorized as “no oxygen”, “oxygen only”, and “ventilation”. In order to handle class imbalance, a Synthetic Minority Oversampling Technique (SMOTE) was applied to the training data. This method addresses the issue of data imbalance by generating synthetic examples of the minority class rather than simply duplicating existing data. To ensure that the LASSO regression results were not influenced by scale differences between variables, all variables included in the study were standardized (mean = 0; standard deviation = 1) prior to analysis. All analyses were performed with R software (4.3.3).

## 3. Results

### 3.1. Data Quality

Of the 124,684 patients that were obtained from the eight healthcare institutions, a number of patients were excluded from the analysis: 32,256 were excluded through the inclusion and exclusion criteria; 23,932 were excluded through the full removal of two healthcare institutions due to missing predictors of interest; and 15,906 were removed through complete case analysis, which removed patients with any missing record across the included predictors. This left a final cohort of 52,590 COVID-19 inpatients (Figure 1).

### 3.2. Data Utility and Variable Selection

Using the LASSO regression model, 16 of 85 variables were eliminated from the model. Full results for 28-day mortality are provided in Table 2. Null values (“.”) indicate a measurement that was eliminated from the model, while non-null coefficients indicate variables that were selected. Reference categories for categorical variables are provided in parentheses.

Among demographics, comorbidities, and indicators of disease severity, the variables that contributed most to prediction of death were need for ventilation (2.054) and need for any oxygen (0.802) compared to no oxygen needed, presence of chronic kidney disease (0.850), higher mean heart rate (0.356), higher mean respiratory rate (0.351), and chronic lung disease (0.350). Higher age groups also showed progressively higher coefficients related to prediction of death, compared to the age group of 18–39, with 40–49 at 0.283, 50–59 at 0.803, 60–69 at 1.619, 70–79 at 2.167, and 80 or more years of age at 2.821. The variables that contributed most to prediction of survival were Non-Hispanic Black race/ethnicity (−0.685), higher mean Sp02 (−0.745), higher minimum temperature (−0.128), and higher minimum respiratory rate (−0.123).

In the case of laboratory measurements, the mean value was dropped from the model for 8 of 13 variables. The most predictive values for mortality were all higher minimum values: higher minimum hematocrit level (0.482), higher minimum leukocyte count (0.348), higher minimum AST level (0.315), and higher minimum total bilirubin (0.236). The most predictive of survival were higher mean hemoglobin level (−0.484), higher mean eosinophil count (−0.448), higher minimum platelet count (−0.418), higher maximum eGFR (−0.416), higher minimum hemoglobin level (−0.302), and higher median ALT level (−0.225).

**Figure 2:**
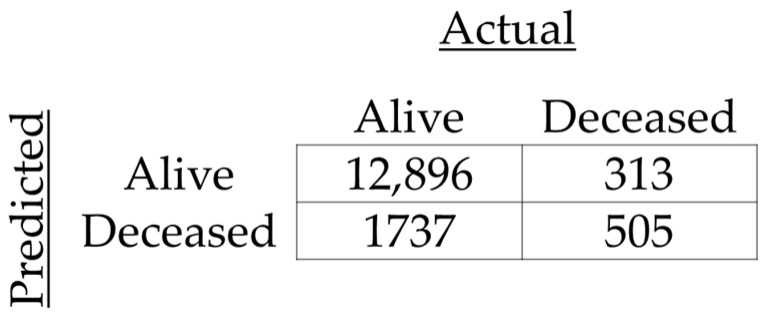
Confusion matrix for prediction of 28-day mortality.

**Figure 3:**
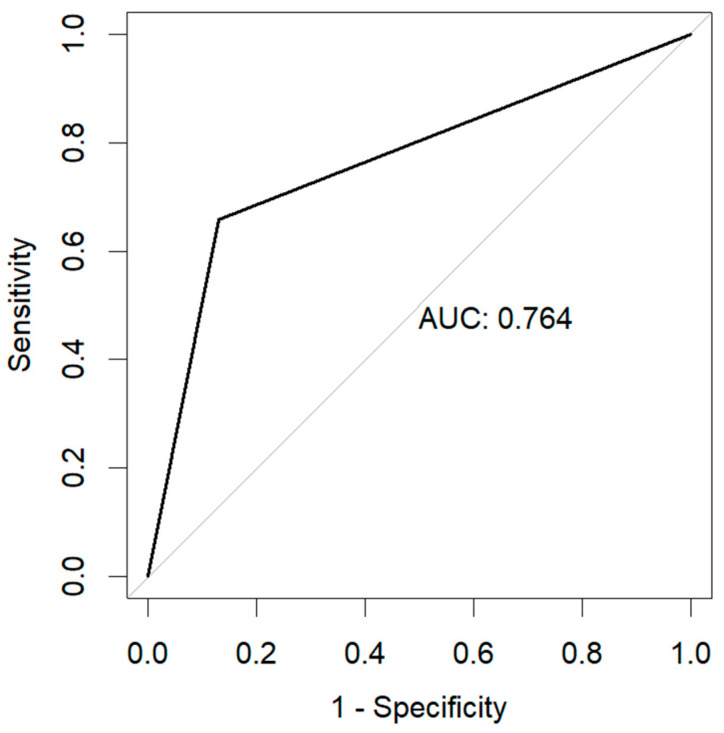
Receiver Operator Characteristic Curve (ROC) as a result of the prediction of 28-day mortality.

The resulting model obtained an accuracy of 0.87, with an F1-score of 0.93 (precision of 0.97 and recall of 0.88), as well as an area under the curve (AUC) of 0.76. The resulting confusion matrix is shown in Figure 2, and the resulting Receiver Operator Characteristic (ROC) curve is shown in Figure 3.

## 4. Discussion

This study aimed to use a data-driven method to assess the usefulness of collaborative data repositories, such as CURE ID, for aiding researchers in understanding risk factors and in identifying treatments for difficult-to-treat infectious diseases. Through the use of a LASSO regression and data from six US healthcare systems, the study assessed the validity of such data and examined the accuracy of prognostic variables for predicting all-cause mortality within 28 days of hospitalization. As evidence of the validity of the data and model, the LASSO retained variables recognized as factors that are associated with risk of 28-day all-cause mortality. The model also achieved high accuracy at 86.7% with 69 variables included, along with an F1-score of 0.93. In the context of medical prognostics, the decision threshold of the LASSO regression model currently limits the number of false positives; however, the main purpose of evaluating the output of the LASSO regression was to validate the resulting factor selection. In light of the need for data and databases designed for collaboration and data sharing, the study demonstrated the utility of such a dataset from which variable selection methods, such as LASSO, can streamline original and drug-repurposing research.

The variables that were retained in the LASSO regression were among those known as risk and protective factors for mortality. As expected, age and indicators of the severity of illness were among the most predictive of mortality [29,30]. Being in a younger age group and not needing oxygen decreased the likelihood of death, as did higher mean oxygen saturation. In contrast, being in an older group, needing ventilation, having higher mean heart and respiratory rates, and having comorbidities increased the likelihood of death. These findings are also consistent with an earlier use of LASSO regression for prognostic variables for COVID-19 using 1154 patients in Wuhan, China [26]. The findings of our study extend this and similar research [27] to 52,590 patients across six US healthcare systems. Therefore, in combination with the data-driven, collaborative nature of the methodology and expanded examination of laboratory value measures, this study addresses persistent data-based hinderances to streamlined research approaches, such a need for more data from diverse settings and sources. The variables identified here can provide a departure point for relevant prognostic variables in future research and clinical assessment, while also demonstrating the utility of data such as these for real-world applications.

The LASSO regression was chosen for this analysis due to several strengths [25] that relate to needs for collaboration and data access for newly emerging and difficult-to-treat diseases for which drug repurposing may be the most effective use of resources. Notably, the LASSO is useful when there is little a priori knowledge about relevant prognostic variables. As new infectious diseases with pandemic potential become more evident in the coming years, methodology that can take any known information and provide a model of variables for clinical outcomes, such as mortality, is useful for early detection of key prognostic factors. Furthermore, selection of the strongest prognostic variables may aid in linking these variables and known characteristics of existing approved or shelved drugs, facilitating drug-repurposing efforts. Similarly, LASSO regression reduces overfitting, making it particularly useful for extending as new data become available, such as with newly emerging diseases. Additionally, LASSO minimizes prediction error, suggesting that the model may outperform other data analysis tools or procedures. Collectively, variable-selection tools, applied to complex, open-source, and collaborative datasets, may aid in mitigating data-quality issues that have persisted in both original research and drug-repurposing research by utilizing a data-driven approach to streamlining research.

This study has several limitations. First, despite our study including many variables and different measures of these variables, there are likely other variables of importance to predicting mortality that could not be included in our analysis. For example, the study may have benefited from being able to include additional comorbidities, such as chronic obstructive pulmonary disease and immunosuppression beyond human immunodeficiency virus. Second, our LASSO regression has less accuracy than a similar study in the literature [26]. However, our study also had more patients and is more comprehensive in the number of variables, which may be inflating variability. Additionally, data collection from real-world sources is subject to potential biases, including inconsistencies in how data are recorded across health systems, and missing data from some patient groups who may have varied levels of access to healthcare. A notable limitation involved the exclusion of two healthcare institutions from the analysis. We intended to include data from eight healthcare institutions, but two institutions were excluded due to missing data mechanisms. Specifically, missingness completely at random (MCAR) was observed in these institutions because of a technical error that prevented sharing the body temperature variable. Furthermore, missing not at random (MNAR) was present for certain variables across all institutions, likely related to the patient’s clinical course, such as laboratory values that were not measured when a patient’s clinical trajectory precluded their collection. For example, patients with less severe illness may have been less likely to have a complete metabolic panel ordered and therefore may be missing liver enzyme values (i.e., total bilirubin, aspartate aminotransferase, and alanine aminotransferase). These missingness mechanisms represent an inherent limitation in the study design, as the technical error and the dependence of data availability on patient outcomes could affect the generalizability of the findings. Future studies should address these issues to improve data completeness and robustness. Finally, the LASSO regression only removed 15 variables, retaining 69 predictors. Examining 69 different measures from a patient may not be practical. Therefore, given that concerning conditions and laboratory values for mortality in COVID-19 are well-established, the results of this study are most useful as a demonstration of the utility of such data which can be rapidly mobilized to inform research in the event of a newly emerging infectious disease or COVID-19 variants with different clinical properties.

## 5. Conclusions

Newly emerging infectious diseases, particularly those with pandemic potential, put tremendous pressure on healthcare systems and on researchers to rapidly develop methodologies to combat disease. Given that there will likely be more incidences of these diseases in upcoming years, systems that streamline data-driven approaches for such research are critical. Among persistent data-quality issues, such as unreliable data sources and inefficient data sharing, this study showed the utility of an open-source, internet-based dataset, CURE ID, for examining prognostic variables. It also demonstrated the use of variable-selection methods to further investigate variables, which can be used to facilitate the reduction of the dataset to key variables and for modeling clinical outcomes (such as 28-day all-cause mortality) as more data become available within collaborative and ongoing research. Therefore, methodologies such as those described here offer considerations for using data-driven approaches for real-world applications to future original and drug-repurposing research. Future applications of these methodologies could extend to other infectious diseases, such as sepsis, where identifying key prognostic variables can improve clinical outcomes and inform treatment strategies.

## Figures and Tables

**Table 1:** Predictors included in the LASSO regression model.

Factors *		Value
Demographics		
Age		66 (52–77)
Race and ethnicity	White	33,986 (64.6)
	Black	8172 (15.6)
	Hispanic	2040 (3.9)
	Other	8392 (15.9)
Sex	Male	27,560 (52.4)
	Female	25,030 (47.6)
Body mass index		29 (24.5–34.7)
Comorbidities	Human immunodeficiency virus	388 (0.73)
	Chronic lung disease	15,655 (29.7)
	Chronic kidney disease	14,898 (28.3)
	Cardiovascular disease	14,769 (28)
	Diabetes	21,529 (40.9)
Indicators of Disease Severity		
Oxygen support	No oxygen	33,369 (63.5)
	Oxygen only	16,761 (31.8)
	Ventilation	2460 (4.7)
Oxygen saturation (%)	Minimum	90.0 (86.0–93.0)
	Median	96.0 (94.0–95.8)
	Mean	95.5 (94.1–97.0)
	Maximum	100.0 (98.0–100.0)
Respiratory rate	Minimum	16.0 (14.0–16.0)
	Median	18.0 (18.0–20.0)
	Mean	19.0 (17.8–21.1)
	Maximum	25.0 (21.0–31.0)
Heart rate	Minimum	64.0 (57.0–72.0)
	Median	81.0 (72.0–90.5)
	Mean	81.7(73.1–91.0)
	Maximum	103.0 (92.0–117.0)
Temperature (F)	Minimum	97.3 (96.8–97.6)
	Median	98.1 (97.8–98.5)
	Mean	98.2 (97.8–98.6)
	Maximum	99.5 (98.8–100.8)
Laboratory Measurements		
Leukocyte count (×10^9^/L)	Minimum	5.7 (4.1–8.0)
	Median	6.9 (5.0–9.5)
	Mean	7.0 (5.1–9.7)
	Maximum	8.2 (6.0–11.6)
Monocyte count (×10^9^/L)	Minimum	0.4 (0.2–0.6)
	Median	0.5 (0.4–0.8)
	Mean	0.5 (0.1–0.9)
	Maximum	0.6 (0.4–0.9)
Lymphocyte count (×10^9^/L)	Minimum	0.8 (0.5–1.2)
	Median	1.0 (0.7–1.4)
	Mean	1.0 (0.7–1.4)
	Maximum	1.16 (0.8–1.69)
Eosinophil count (×10^9^/L)	Minimum	0 (0–0.01)
	Median	0 (0–0.05)
	Mean	0 (0–0.06)
	Maximum	0 (0–0.07)
Basophil count (×10^9^/L)	Minimum	0 (0–0.01)
	Median	0 (0–0.03)
	Mean	0.01 (0–0.06)
	Maximum	0.01 (0–0.19)
Hematocrit level (%)	Minimum	36.1 (31.5–40.0)
	Median	37.8 (33.3–41.6)
	Mean	38.0 (33.5–41.7)
	Maximum	40.0 (35.5–43.8)
Hemoglobin level (g/L)	Minimum	11.8 (10.1–13.2)
	Median	12.4 (10.7–13.7)
	Mean	12.4 (10.8–13.8)
	Maximum	13.1 (11.5–14.5)
Platelet count (×10^9^/L)	Minimum	190.0 (144.0–248.0)
	Median	206.0 (157.0–268.0)
	Mean	208.3 (159.0–270.7)
	Maximum	228.0 (174.0–298.0)
Total bilirubin	Minimum	0.4 (0.3–0.6)
	Median	0.5 (0.4–0.7)
	Mean	0.5 (0.2–0.9)
	Maximum	0.6 (0.4–0.8)
Aspartate aminotransferase level (U/L)	Minimum	29.0 (20.0–44.0)
	Median	33.0 (23.0–52.0)
	Mean	33.5 (23.0–53.0)
	Maximum	38.0 (25.0–61.0)
Alanine aminotransferase level (U/L)	Minimum	25.0 (16.0–40.0)
	Median	27.0 (18.0–45.0)
	Mean	27.5 (18.0–45.7)
	Maximum	30.0 (19.0–51.0)
Serum creatinine level (mg/dL)	Minimum	0.9 (0.7–1.2)
	Median	0.9 (0.7–1.3)
	Mean	0.9 (0.7–1.3)
	Maximum	1.1 (0.8–1.5)
Estimated glomerular filtration rate	Minimum	65.0 (39.4–88.4)
	Median	75.0 (47.0–95.1)
	Mean	74.3 (47.3–94.1)
	Maximum	82.5 (54.3–76.9)
Outcome		
Mortality	Alive	48,673 (92.6)
	Deceased	3917 (7.4)

* Continuous variables are presented as median (interquartile range), and categorical variables are presented as counts (percentages).

**Table 2:** Results from LASSO regression model.

Variable		Coefficient
Demographics		
Age (18–39 years)	40–49 years	0.283
	50–59 years	0.803
	60–69 years	1.619
	70–79 years	2.167
	80+ years	2.821
Race and ethnicity (White)	Black	−0.685
	Hispanic	−0.032
	Other	0.021
Sex (female)		0.252
Body mass index		−0.214
Comorbidities (lack of presence of condition)	Human immunodeficiency virus	0.276
	Chronic lung disease	0.350
	Chronic kidney disease	0.850
	Cardiovascular disease	0.139
	Diabetes	0.093
Indicators of Disease Severity		
Oxygen support (no oxygen)	Oxygen only	0.802
	Ventilation	2.054
Oxygen saturation (%)	Minimum	−0.046
	Median	0.153
	Mean	−0.745
	Maximum	0.292
Respiratory rate	Minimum	−0.123
	Median	0.076
	Mean	0.351
	Maximum	−0.019
Heart rate	Minimum	−0.075
	Median	−0.065
	Mean	0.356
	Maximum	−0.083
Temperature (F)	Minimum	−0.128
	Median	0.077
	Mean	
	Maximum	0.014
Laboratory Measurements		
Leukocyte count	Minimum	0.348
	Median	
	Mean	
	Maximum	0.001
Monocyte count	Minimum	0.064
	Median	−0.115
	Mean	
	Maximum	−0.064
Lymphocyte count	Minimum	−0.045
	Median	−0.079
	Mean	−0.037
	Maximum	
Eosinophil count	Minimum	−0.107
	Median	0.202
	Mean	−0.448
	Maximum	−0.089
Basophil count	Minimum	−0.098
	Median	0.054
	Mean	−0.081
	Maximum	−0.111
Hematocrit level	Minimum	0.482
	Median	0.055
	Mean	
	Maximum	0.040
Hemoglobin level	Minimum	−0.302
	Median	0.070
	Mean	−0.484
	Maximum	
Platelet count	Minimum	−0.418
	Median	0.075
	Mean	
	Maximum	
Total bilirubin	Minimum	0.236
	Median	
	Mean	
	Maximum	−0.091
Aspartate aminotransferase level (U/L)	Minimum	0.315
	Median	
	Mean	0.087
	Maximum	0.066
Alanine aminotransferase level (U/L)	Minimum	−0.173
	Median	−0.225
	Mean	
	Maximum	0.081
Serum creatinine level (mg/dL)	Minimum	0.125
	Median	
	Mean	
	Maximum	−0.188
Estimated glomerular filtration rate	Minimum	0.136
	Median	0.129
	Mean	
	Maximum	−0.416

## Data Availability

The data presented in this study are available upon request from the corresponding author.
